# Reduced Incidence of Foot-Related Hospitalisation and Amputation amongst Persons with Diabetes in Queensland, Australia

**DOI:** 10.1371/journal.pone.0130609

**Published:** 2015-06-22

**Authors:** Peter A. Lazzarini, Sharon R. O’Rourke, Anthony W. Russell, Patrick H. Derhy, Maarten C. Kamp

**Affiliations:** 1 School of Clinical Sciences, Queensland University of Technology, Brisbane, Queensland, Australia; 2 Allied Health Research Collaborative, Metro North Hospital & Health Service, Queensland Health, Brisbane, Queensland, Australia; 3 Cairns Diabetes Centre, Queensland Health, Cairns, Queensland, Australia; 4 Department of Diabetes & Endocrinology, Princess Alexandra Hospital, Brisbane, Queensland, Australia; 5 School of Medicine, The University of Queensland, Brisbane, Queensland, Australia; 6 Clinical Access and Redesign Unit, Queensland Health, Brisbane, Queensland, Australia; 7 Diabetes Australia (Queensland), Brisbane, Queensland, Australia; Heinrich-Heine University, Faculty of Medicine, GERMANY

## Abstract

**Objective:**

To determine trends in the incidence of foot-related hospitalisation and amputation amongst persons with diabetes in Queensland (Australia) between 2005 and 2010 that coincided with changes in state-wide ambulatory diabetic foot-related complication management.

**Methods:**

All data from cases admitted for the principal reason of diabetes foot-related hospitalisation or amputation in Queensland from 2005–2010 were obtained from the Queensland Hospital Admitted Patient Data Collection dataset. Incidence rates for foot-related hospitalisation (admissions, bed days used) and amputation (total, minor, major) cases amongst persons with diabetes were calculated per 1,000 person-years with diabetes (diabetes population) and per 100,000 person-years (general population). Age-sex standardised incidence and age-sex adjusted Poisson regression models were also calculated for the general population.

**Results:**

There were 4,443 amputations, 24,917 hospital admissions and 260,085 bed days used for diabetes foot-related complications in Queensland. Incidence per 1,000 person-years with diabetes decreased from 2005 to 2010: 43.0% for hospital admissions (36.6 to 20.9), 40.1% bed days (391 to 234), 40.0% total amputations (6.47 to 3.88), 45.0% major amputations (2.18 to 1.20), 37.5% minor amputations (4.29 to 2.68) (*p* < 0.01 respectively). Age-sex standardised incidence per 100,000 person-years in the general population also decreased from 2005 to 2010: 23.3% hospital admissions (105.1 to 80.6), 19.5% bed days (1,122 to 903), 19.3% total amputations (18.57 to 14.99), 26.4% major amputations (6.26 to 4.61), 15.7% minor amputations (12.32 to 10.38) (*p* < 0.01 respectively). The age-sex adjusted incidence rates per calendar year decreased in the general population (rate ratio (95% CI)); hospital admissions 0.949 (0.942–0.956), bed days 0.964 (0.962–0.966), total amputations 0.962 (0.946–0.979), major amputations 0.945 (0.917–0.974), minor amputations 0.970 (0.950–0.991) (*p* < 0.05 respectively).

**Conclusions:**

There were significant reductions in the incidence of foot-related hospitalisation and amputation amongst persons with diabetes in the population of Queensland over a recent six-year period.

## Introduction

Foot-related complications are one of the most common reasons for hospitalisation and lower extremity amputation amongst persons with diabetes [1–4]. Furthermore, people hospitalised with diabetes and foot-related complications experience longer lengths of hospital stay, higher hospital costs and higher mortality rates compared to those hospitalised with diabetes without foot-related complications [4–7]. The effect of foot-related complications on individuals with diabetes can also have profound ongoing implications on physical function, mental health and quality of life [8–11].

Amputation rates amongst persons with diabetes have been suggested as an important quality indicator of health system performance in geographical regions [12–16]. Reducing the incidence of amputations amongst persons with diabetes indicates effective system-wide health care of diabetes and diabetes complications [12–16]. These recommendations are based on studies demonstrating reductions in amputations when integrated evidence-based multi-faceted foot-related complication management strategies are delivered amongst persons with diabetes in defined geographical regions [16–21].

Australia has been reported to have high amputation rates amongst persons with diabetes (approximately 18 per 100,000 person-years of the general population) in comparison to other developed nations (median of approximately 12 per 100,000 person-years) [4, 22, 23]. Whilst it is acknowledged that amputation rate comparisons between different regions or nations are challenging, due to methodological differences in data sources and numerator and denominator definitions [3, 12, 15, 16], further reports indicate that Australia’s national amputation rates amongst persons with diabetes were increasing [7, 24]. However, a study of Australian national or regional amputation rates amongst persons with diabetes over time has not been performed since the 1990s [25]. The last study analysed rates between 1995 and 1998 and reported a relatively steady rate of 14 diabetes-related amputations per 100,000 person-years in the general population during this period [25].

The state of Queensland is the second largest Australian state in terms of geographical area, third largest in population and most decentralised state incorporating extremely diverse geography and demography [26] which makes systematic delivery of health care challenging. Thus, Queensland appears to be an appropriate representational regional population to inform foot-related hospitalisation and amputation rate trends amongst persons with diabetes in Australia. Moreover the Queensland Statewide Diabetes Clinical Network commissioned a *Diabetic Foot Innovation Project* to address the significant growing burden of diabetes foot-related complications in Queensland in 2007 [27]. In 2008 the project implemented multi-faceted evidence-based strategies in geographically diverse pilot regions to improve diabetes foot-related complication management in ambulatory services in an attempt to reduce foot-related hospitalisation and amputations amongst persons with diabetes [27]. Subsequently these strategies were rolled out to cover most health regions in Queensland by 2010 [28].

Thus, this study’s primary aim was to determine the annual incidence of foot-related hospitalisation and amputation amongst persons with diabetes in Queensland (Australia) between 2005 and 2010. The secondary aim was to observe trends pre- and post-implementation of staged statewide ambulatory diabetes foot-related complication service strategies in Queensland.

## Methods

### Ethics Statement

This study was a retrospective analysis of a population-based hospital discharge dataset (Queensland Hospital Admitted Patient Data Collection (QHAPDC) [29]). The Human Research Ethics Committee at The Prince Charles Hospital, Brisbane, Australia provided ethical approval for the study (Ethics No. HREC/12/QPCH/8) and the Office of Health and Medical Research, Queensland Government, Australia provided approval to access anonymised QHAPDC data for the purposes of the study. All QHPADC data obtained for the purposes of this study was anonymised by the Health Statistics Branch (Queensland Government) prior to providing it to the authors for analysis. Thus, individual patient consent was not required or available.

### Data source

The QHAPDC dataset covers all public and licensed private inpatient and day hospital admission separation activity in the state of Queensland, Australia [29]. All patients discharged between 1^st^ January 2005 and 31^st^ December 2010 from a Queensland hospital for the principal reason of a diabetes foot-related complication or amputation procedure were identified through a series of 32 International Classification of Diseases, Tenth Revision, Australian Modification (ICD-10-AM, Fifth Edition) diagnoses codes and twelve ICD-10-AM lower extremity amputation procedural codes (Table 1).

**Table 1:** ICD codes (ICD-10-AM) used to identify a principal diagnosis of a diabetes foot-related complication.

Diabetes foot-related complication	ICD Codes	Comments
**Principal diagnosis**		
Peripheral Neuropathy	E1042, E1142, E1342, E1442	
Peripheral Vascular Disease (with and without gangrene)	E1051, E1052, E1151, E1152, E1351, E1352, E1451, E1452	
Foot Ulcer	E1073, E1173, E1373, E1473	
Other specified skin and subcutaneous tissue complication	E1062, E1162, E1362, E1462	
Specific diabetic musculoskeletal & connective tissue complications	E1061, E1161, E1361, E1461	
Cellulitis (of toe)	LO302	Only if additional diagnosis diabetes code present (i.e. E10 –E14)
Cellulitis (of lower limb)	L0311	Only if additional diagnosis diabetes code present (i.e. E10 –E14)
Charcots	M146	Only if additional diagnosis diabetes code present (i.e. E10 –E14)
Acute / sub-acute osteomyelitis	M8617, M8627	Only if additional diagnosis diabetes code present (i.e. E10 –E14)
Chronic osteomyelitis	M8647, M8667	Only if additional diagnosis diabetes code present (i.e. E10 –E14)
Other osteomyelitis	M8697	Only if additional diagnosis diabetes code present (i.e. E10 –E14)
**Procedure**		
Foot / ankle amputation (Minor Amputation)	[1533] 4433800, 4435800, 9055700, 4436100, 4436101, 4436400, 4436401	Only if additional diagnosis diabetes code present (i.e. E10 –E14)
Below knee amputation (Major Amputation)	[1505] 4436701, 4436702	Only if additional diagnosis diabetes code present (i.e. E10 –E14)
Above knee amputation (Major Amputation	[1484] 4437000, 4437300, 4436700	Only if additional diagnosis diabetes code present (i.e. E10 –E14)

### Outcome definitions

The outcomes, used as the numerators for this study, were cases of foot-related hospitalisation (admissions and occupied bed days used) and amputations (total, minor and major) amongst persons with diabetes. Diabetes foot-related hospitalisations were defined as having diabetes and a foot-related complication diagnosed and entered in the hospital medical record by a physician during the hospital separation, and subsequently coded and entered in the QHAPDC by a hospital coder [29]. Diabetes foot-related hospital admission cases were identified from the QHAPDC dataset as any hospital separation discharge with a principal diagnoses or procedural code outlined in Table 1. A hospital separation is defined as ending at discharge, transfer, death or major change in episode of care type [29]. Hospital occupied bed days used were defined as the overall number of bed days used during the length of stay for the above defined diabetes foot-related hospital admission cases. Average length of stay was calculated using the numerator of total number of hospital occupied bed days used over the denominator of total number of hospital admission cases for each calendar year.

Amputation cases were defined as any lower extremity amputation procedural code identified from the above defined foot-related hospital admissions amongst persons with diabetes (Table 1). Major amputations were defined as all procedural codes for lower extremity amputation procedures through or proximal to the ankle, and, minor amputations were amputation procedures distal to the ankle. In admissions recording multiple amputation procedure codes for the same admission separation, the highest level of amputation was assigned as the single amputation procedure for that admission only. All amputation procedures with an additional diagnosis code of diabetes were included. Thus, amputation cases that may have been primarily related to other causes such as malignancy or trauma were included in this analysis. Data extracted for each hospital admission case included age, sex, length of stay, year of discharge and any aforementioned identified diabetes foot-related principal diagnoses or amputation procedural codes. Ages younger than 18 years and older than 85 years were grouped into ‘<18 years’ and ‘>85 years’ groups respectively by the Health Statistics Branch prior to providing data to the authors to ensure that the potentially small number of people in these groups were not re-identifiable by the authors or other third parties.

### Population definitions

Two different populations were used as different denominators to determine the incidence rates for all outcomes as recommended [12, 15–17]. These populations were the general population and diabetes (‘at risk’) population of Queensland for each calendar year from 2005 to 2010. The general population numbers, including age and sex category numbers, were obtained directly from the official Australian Bureau of Statistics midyear general resident population numbers for the state of Queensland (Australia) for each calendar year from 2005 to 2010 [30]. The diabetes population numbers for Queensland were not publicly available for the six-year period from 2005 to 2010. Queensland diabetes population numbers for each calendar year were therefore estimated using data from the Australian National Diabetes Services Scheme (NDSS) register [31, 32]. This was performed using the proportion of the number of people registered with diagnosed diabetes on the NDSS register in Queensland (199,242) in the most recent reported year of 2013 compared with those registered with diagnosed diabetes in Australia (1,093,125) from the same source and year [32]. The Queensland proportion from 2013 (18.23%) [32] was then multiplied by the available midyear numbers of people registered with diagnosed diabetes on the NDSS register in Australia for each calendar year to determine an estimated midyear diabetes population in Queensland for the years 2005 to 2010 [31]. Age and sex category numbers for the Queensland diabetes population were not available and unable to be estimated [31, 32].

### Incidence rate definitions

The incidence rates for this study were determined using the formula of the number of outcome cases (numerator) over the number of population in person-years (denominator) for the calendar year [33]. Specific incidence rates determined for this study were: 1) hospital admission cases due to diabetes-related foot complications per 100,000 person-years (general population); 2) hospital admission cases due to diabetes-related foot complications per 1,000 person-years with diabetes (diabetes population); 3) hospital bed days used due to diabetes-related foot complications per 100,000 person-years (general population); 4) hospital bed days used due to diabetes-related foot complications per 1,000 person-years with diabetes (diabetes population); 5) total amputation cases from any cause in people with diabetes per 100,000 person-years (general population); 6) total amputation cases from any cause in people with diabetes per 1,000 person-years with diabetes (diabetes population); 7) minor amputation cases from any cause in people with diabetes per 100,000 person-years (general population); 8) minor amputation cases from any cause in people with diabetes per 1,000 person-years with diabetes (diabetes population); 9) major amputation cases from any cause in people with diabetes per 100,000 person-years (general population); and, 10) major amputation cases from any cause in people with diabetes per 1,000 person-years with diabetes (diabetes population).

### Data analysis

Data was analysed using SPSS 22.0 for Windows (SPSS Inc., Chicago, IL, USA) and Microsoft Excel 2010. Descriptive statistics were used to display absolute numbers and proportions for each outcome per calendar year, stratified by sex and age categories (0–34, 35–54, 55–74 and >75 years). Median age (interquartile ranges) was also calculated for each outcome per calendar year. Chi-squared tests of independence were used to test differences in sex and age category proportions for each outcome between calendar years. Kruskal-Wallis tests were used to test differences in median age and average length of stay for each outcome between calendar years.

Crude annual incidence rates were calculated for all ten aforementioned defined incidence rates [33]. Age-sex adjusted annual incidence rates using a direct standardized method were also calculated for hospitalisations (admissions and bed days) and amputations (total, minor and major) for the general population [33]. The 2006 mid-year general resident population of Queensland (Australia) was used as the referent year for the age-sex standardization as this was the official year of the Australian census count by the Australian Bureau of Statistics [30]. All age-sex standardized incidences for each calendar year were adjusted based on the age and sex distribution of the 2006 calendar year’s population [30] and expressed per 100,000 person-years of the general population [33]. Age-sex adjusted annual incidence rates for the diabetes population were unable to be calculated using available data [31, 32]. Confidence intervals (95% CI) were calculated for the crude and age-sex adjusted annual incidences for the overall outcomes of hospitalisation (admissions and bed days) and amputations (total, minor and major), using the Wilson score method without continuity correction [33]. Crude incidence rates for all outcomes were also calculated separately for each sex and age category in the general population. Chi-squared test of trend were used to assess changes in incidence over time for each sex and age category for all outcomes.

To more robustly test for time trends Poisson regression models were fitted for hospitalisation (admission and bed days) and amputation (total, minor and major), rates across the six year period offset by the Queensland general population. Three models were used for each outcome; the first including calendar year only, the second adjusting for the independent variables of sex and age categories and the third model adjusting for age, sex and the interaction variables of sex*age categories. Calendar year was treated and reported both as a categorical and continuous variable to evaluate the robustness of findings.

## Results

Table 2 displays the absolute numbers of foot-related hospitalisations (admissions and bed days) and amputations (total, minor and major) cases amongst persons with diabetes for each calendar year, along with the general resident population and estimated diabetes population of Queensland. There were 24,917 hospital admissions for the principal management of a diabetes foot-related complication between 2005 and 2010 resulting in the use of 260,085 hospital occupied bed days. These included 4,443 hospital admissions for amputation procedures amongst persons with diabetes occurring during this six-year period; major amputations made up 32% (1,434) and minor amputations 68% (3,009).

**Table 2:** Absolute numbers of foot-related hospitalisation (admissions, bed days and average length of stay (ALOS)) and amputation (total, minor and major) cases amongst persons with diabetes, plus, estimated diabetes and general resident population, in Queensland from 2005 to 2010.

Variables	2005	2006	2007	2008	2009	2010	Total	*p* Value^
**Hospitalisations**								
**Admissions**	4,082	4,463	4,257	4,427	4,047	3,641	24,917	
Sex								
Female	1,337 (32.8%)	1,441 (32.3%)	1,422 (33.4%)	1,527 (34.5%)	1,321 (32.6%)	1,189 (32.7%)	8,237 (33.1%)	
Male	2,745 (67.2%)	3,022 (67.7%)	2,835 (66.6%)	2,900 (65.5%)	2,726 (67.4%)	2,452 (67.3%)	16,680 (66.9%)	0.278
Median Age (IQR)#	67(58–76)	67(58–76)	66(57–76)	66(58–76)	67(58–77)	67(58–76)	67(58–76)	0.105
Age category								
0–34 years	56 (1.4%)	127 (2.8%)	89 (2.1%)	91 (2.1%)	70 (1.7%)	85 (2.3%)	518 (2.1%)	
35–54 years	694 (17.0%)	699 (15.7%)	739 (17.4%)	787 (17.8%)	679 (16.8%)	617 (16.9%)	4,215 (16.9%)	
55–74 years	2,169 (53.1%)	2,354 (52.7%)	2,209 (51.9%)	2,276 (51.4%)	2,087 (51.6%)	1,913 (52.5%)	13,008 (52.2%)	
>75 years	1,163 (28.5%)	1,283 (28.7%)	1,220 (28.7%)	1,273 (28.8%)	1,211 (29.9%)	1,026 (28.2%)	7,176 (28.8%)	0.001*
**Bed Days**	43,564	42,819	44,634	44,944	43,300	40,824	260,085	
Sex								
Female	16,077 (36.9%)	13,901 (32.5%)	15,403 (34.5%)	15,123 (33.6%)	13,767 (31.8%)	13,632 (33.4%)	87,903 (33.8%)	
Male	27,487 (63.1%)	28,918 (67.5%)	29,231 (65.5%)	29,821 (66.4%)	29,533 (68.2%)	27,192 (66.6%)	172,182 (66.2%)	<0.001*
Age category								
0–34 years	321 (0.7%)	668 (1.6%)	777 (1.7%)	863 (1.9%)	678 (1.6%)	916 (2.2%)	4,223 (1.6%)	
35–54 years	8,087 (18.6%)	7,778 (18.2%)	7,330 (16.4%)	7,374 (16.4%)	7,533 (17.4%)	6,520 (16.0%)	44,622 (17.2%)	
55–74 years	20,798 (47.7%)	20,088 (46.9%)	22,814 (51.1%)	22,168 (49.3%)	21,463 (49.6%)	21,141 (51.8%)	128,472 (49.4%)	
>75 years	14,358 (33.0%)	14,285 (33.4%)	13,713 (30.7%)	14,539 (32.3%)	13,626 (31.5%)	12,247 (30.0%)	82,768 (31.8%)	<0.001*
**ALOS** #	10.7	9.6	10.5	10.2	10.7	11.2	10.4	<0.001
Sex#								
Female	12.0	9.6	10.8	9.9	10.4	11.5	10.7	
Male	10.0	9.6	10.3	10.3	10.8	11.1	10.3	<0.001
Age category#								
0–34 years	5.7	5.3	8.7	9.5	9.7	10.8	8.2	
35–54 years	11.7	11.1	9.9	9.4	11.1	10.6	10.6	
55–74 years	9.6	8.5	10.3	9.7	10.3	11.1	9.9	
>75 years	12.3	11.1	11.2	11.4	11.3	11.9	11.5	<0.001
**Amputations**								
**Total**	721	759	776	754	756	677	4,443	
Sex								
Female	238 (33.0%)	236 (31.1%)	238 (30.7%)	211 (28.0%)	196 (25.9%)	196 (29.0%)	1,315 (29.6%)	
Male	483 (67.0%)	523 (68.9%)	538 (69.3%)	543 (72.0%)	560 (74.1%)	481 (71.0%)	3,128 (70.4%)	0.047*
Median Age (IQR)#	66(56–76)	67(57–77)	66(57–76)	66(56–76)	66(57–76)	67(58–75)	66(56–76)	0.256
Age category								
0–34 years	4 (0.6%)	12 (1.6%)	14 (1.8%)	17 (2.3%)	16 (2.1%)	12 (1.8%)	75 (1.7%)	
35–54 years	146 (20.2%)	145 (19.1%)	139 (17.9%)	142 (18.8%)	142 (18.8%)	121 (17.9%)	835 (18.8%	
55–74 years	365 (50.6%)	369 (48.6%)	388 (50.0%)	391 (51.9%)	383 (50.7%)	364 (53.8%)	2,260 (50.9%)	
>75 years	206 (28.6%)	233 (30.7%)	235 (30.3%)	204 (27.1%)	215 (28.4%)	180 (26.6%)	1,273 (28.7%)	0.449
**Minor**	478	503	520	530	510	468	3,009	
Sex								
Female	147 (30.8%)	156 (31.0%)	157 (30.2%)	141 (26.6%)	128 (25.1%)	134 (28.6%)	863 (28.7%)	
Male	331 (69.2%)	347 (69.0%)	363 (69.8%)	389 (73.4%)	382 (74.9%)	334 (71.4%)	2,146 (71.3%)	0.203
Median Age (IQR)#	64(55–74)	65(56–75)	64(55–74)	64(54–73)	64(55–74)	65(56–74)	64(55–74)	0.261
Age category								
0–34 years	4 (0.8%)	11 (2.2%)	11 (2.1%)	13 (2.5%)	11 (2.2%)	8 (1.7%)	58 (1.9%)	
35–54 years	112 (23.4%)	110 (21.9%)	108 (20.8%)	108 (20.4%)	111 (21.8%)	99 (21.2%)	648 (21.5%)	
55–74 years	251 (52.5%)	248 (49.3%)	268 (51.5%)	290 (54.7%)	268 (52.5%)	252 (53.8%)	1,577 (52.4%)	
>75 years	111 (23.2%)	134 (26.6%)	133 (25.6%)	119 (22.5%)	120 (23.5%)	109 (23.3%)	726 (24.1%)	0.816
**Major**	243	256	256	224	246	209	1,434	
Sex								
Female	91 (37.4%)	80 (31.2%)	81 (31.6%)	70 (31.2%)	68 (27.6%)	62 (29.7%)	452 (32.8%)	
Male	152 (62.6%)	176 (68.8%)	175 (68.4%)	154 (68.8%)	178 (72.4%)	147 (70.3%)	982 (67.2%)	0.304
Median Age (IQR)#	71(62–81)	71(62–81)	71(62–81)	70(61–80)	70(61–80)	70(61–79)	71(62–80)	0.353
Age category								
0–34 years	0	1 (0.4%)	3 (1.2%)	4 (1.8%)	5 (2.0%)	4 (1.9%)	17 (1.2%)	
35–54 years	34 (14.0%)	35 (13.7%)	31 (12.1%)	34 (15.2%)	31 (12.6%)	22 (10.5%)	187 (13.0%)	
55–74 years	114 (46.9%)	121 (47.3%)	120 (46.9%)	101 (45.1%)	115 (46.7%)	112 (53.6%)	683 (47.6%)	
>75 years	95 (39.1%)	99 (38.7%)	102 (39.8%)	85 (37.9%)	95 (38.6%)	71 (34.0%)	547 (38.1%)	0.617
**Population**								
Diabetes Population	111,476	122,073	133,424	145,548	159,954	174,492	846,967	
General Population	3,918,494	4,007,992	4,111,018	4,219,505	4,328,771	4,404,744	24,990,524	
Diabetes Prevalence (%)	2.84%	3.05%	3.25%	3.45%	3.70%	3.96%	3.39%	

Data are number (%); ALOS: average length of stay; IQR: Interquartile range

^ Chi-squared tests of independence used unless otherwise indicated

# Kruskal-Wallis tests used

* *p* < 0.05.

Numbers of hospital admission cases and hospital bed days used for the principal management of diabetes foot-related complications decreased from 4,082 in 2005 to 3,641 in 2010, and, 43,564 in 2005 to 40,824 in 2010, respectively. Conversely average length of stay increased from 10.7 days in 2005 to 11.2 days in 2010 (*p* < 0.01). Total amputation case numbers also decreased from 721 in 2005 to 677 in 2010. This included major amputations from 243 in 2005 to 209 in 2010, and, minor amputations from 478 in 2005 to 468 in 2010. The median (interquartile range) age for cases hospitalised for diabetes foot-related complications was 67(58–76) years and for total amputations was 66(56–76) years; the median age did not change over the six-year period for either outcome (*p* = 0.105 and *p* = 0.256, respectively). The proportion of male cases hospitalised for diabetes foot-related complications was 66.9% and for total amputations was 70.4%; the proportion of male cases hospitalised did not change over the six year period (*p* = 0.278), however the proportion of male total amputation cases did increase from 67.0% in 2005 to 71.0% in 2010 (*p* = 0.047).

Table 3 displays the crude incidence per 100,000 person-years (general population) and per 1,000 person-years with diabetes (diabetes population) for foot-related hospitalisations (admissions and occupied bed days) and amputation (major, minor and total) cases amongst persons with diabetes for each calendar year. The crude incidence in the diabetes population decreased from 2005 to 2010 for all outcomes per 1,000 person-years with diabetes: 43.0% for hospitals admissions (36.6 (35.5–37.7) in 2005 to 20.9 (20.2–21.5) in 2010), 40.1% for hospital bed days used (391 (388–394) in 2005 to 234 (232–236) in 2010), 40.0% for total amputations (6.47 (6.01–6.96) in 2005 to 3.88 (3.60–4.18) in 2010), 45.0% for major amputations (2.18 (1.92–2.47) in 2005 to 1.20 (1.05–1.37) in 2010), and 37.5% for minor amputations (4.29 (3.92–4.69) in 2005 to 2.68 (2.45–2.94) in 2010) (*p* < 0.01 respectively). The crude incidence in the general population also decreased from 2005 to 2010 for all outcomes per 100,000 person-years: 20.6% for hospitals admissions (104.2 (101.0–107.4) in 2005 to 82.7 (80.0–85.4) in 2010), 16.6% for hospital bed days used (1,112 (1,101–1,122) in 2005 to 927 (918–934) in 2010), 16.5% for total amputations (18.40 (17.11–19.79) in 2005 to 15.37 (14.25–16.57) in 2010), 23.5% for major amputations (6.20 (5.47–7.03) in 2005 to 4.74 (4.14–5.43) in 2010), and 13.0% for minor amputations (12.20 (11.15–13.34) in 2005 to 10.62 (9.70–11.63) in 2010) (*p* < 0.01 respectively). Crude incidence rates significantly decreased for all sex and age categories for hospital admissions and total amputations (*p* < 0.05 respectively); with the exception of non-significant decreases for male total amputations and increases for the 0–34 year age category for both hospital admissions and total amputations (*p* > 0.05 respectively).

**Table 3:** Incidence rates of foot-related hospitalisation (admissions and bed days) and amputation (total, minor and major) cases amongst persons with diabetes in the estimated diabetes population (per 1,000 person-years) and general resident population (per 100,000 person-years) in Queensland from 2005 to 2010.

Variables	2005	2006	2007	2008	2009	2010	Six-year change (%)^
**Hospitalisations**							
**Admissions**							
***Diabetes Population***							
Crude Rate (95% CI)	36.6 (35.5–37.7)	36.6 (35.5–37.6)	31.9 (31.0–32.9)	30.4 (29.5–31.3)	25.3 (24.5–26.1)	20.9 (20.2–21.5)	-43.0*
***General Population***							
Crude Rate (95% CI)	104.2 (101.0–107.4)	111.4 (108.1–114.7)	103.6 (100.5–106.7)	104.9 (101.9–108.1)	93.5 (90.7–96.4)	82.7 (80.0–85.4)	-20.6*
Age-Sex Rate (95% CI)#	105.1 (102.0–108.3)	111.4 (108.2–114.7)	102.9 (99.8–106.1)	104.0 (100.9–107.2)	92.4 (89.5–95.4)	80.6 (77.9–83.4)	-23.3*
Sex							
Female	68.1	71.8	69.1	72.3	61.0	53.9	-20.9*
Male	140.5	151.1	138.1	137.6	126.0	111.5	-20.6*
Age category							
0–34 years	2.9	6.6	4.5	4.5	3.35	4.0	+37.9
35–54 years	62.2	61.3	63.2	65.9	55.8	50.2	-19.3*
55–74 years	318.3	333.0	301.2	299.4	265.3	235.3	-26.1*
>75 years	538.9	576.5	534.9	547.5	511.0	423.1	-21.5*
**Bed Days**							
***Diabetes Population***							
Crude Rate (95% CI)	391 (388–394)	351 (348–353)	335 (332–337)	309 (306–311)	271 (269–273)	234 (232–236)	-40.1*
***General Population***							
Crude Rate (95% CI)	1,112 (1,101–1,122)	1,068 (1,058–1,078)	1,086 (1,076–1,096)	1,065 (1,055–1,075)	1,000 (991–1,010)	927 (918–934)	-16.6*
Age-Sex Rate (95% CI)#	1,122 (1,112–1,132)	1,068 (1,059–1,078)	1,078 (1,068–1,087.41)	1,056 (1,046–1,065)	988 (979–997)	903 (894–912)	-19.5*
Sex							
Female	818	692	748	716	636	618	-24.4*
Male	1,407	1,446	1,424	1,415	1,366	1,236	-12.2*
Age category							
0–34 years	17	34	39	42	32	43	+152.9*
35–54 years	725	682	627	617	619	530	-26.9*
55–74 years	3,052	2,842	3,111	2,916	2,729	2,600	-14.8*
>75 years	6,653	6,419	6,012	6,253	5,751	5,050	-24.1*
**Amputations**							
**Total**							
***Diabetes Population***							
Crude Rate (95% CI)	6.47 (6.01–6.96)	6.22 (5.79–6.67)	5.82 (5.42–6.24)	5.18 (4.82–5.56)	4.73 (4.40–5.07)	3.88 (3.60–4.18)	-40.0*
***General Population***							
Crude Rate (95% CI)	18.40 (17.11–19.79)	18.94 (17.64–20.33)	18.88 (17.59–20.25)	17.87 (16.64–19.19)	17.46 (16.26–18.75)	15.37 (14.25–16.57)	-16.5*
Age-Sex Rate (95% CI)#	18.57 (17.28–19.95)	18.94 (17.64–20.33)	18.75 (17.46–20.15)	17.70 (16.42–19.03)	17.25 (16.00–18.56)	14.99 (13.84–16.24)	-19.3*
Sex							
Female	12.12	11.75	11.56	9.99	9.05	8.89	-26.7*
Male	24.72	26.15	26.21	25.77	25.89	21.87	-11.5
Age category							
0–34 years	0.21	0.62	0.71	0.84	0.77	0.57	+171.4
35–54 years	13.09	12.71	11.89	11.89	11.67	9.84	-24.8*
55–74 years	53.56	52.21	52.90	51.44	48.69	44.77	-16.4*
>75 years	95.45	104.69	103.02	87.73	90.73	74.22	-22.2*
**Minor**							
***Diabetes Population***							
Crude Rate (95% CI)	4.29 (3.92–4.69)	4.12 (3.78–4.50)	3.90 (3.58–4.25)	3.64 (3.34–3.96)	3.19 (2.92–3.48)	2.68 (2.45–2.94)	-37.5*
***General Population***							
Crude Rate (95% CI)	12.20 (11.15–13.34)	12.55 (11.50–13.70)	12.65 (11.60–13.78)	12.56 (11.54–13.68)	11.78 (10.80–12.85)	10.62 (9.70–11.63)	-13.0*
Age-Sex Rate (95% CI)#	12.32 (11.28–13.46)	12.55 (11.50–13.69)	12.58 (11.52–13.72)	12.44 (11.39–13.57)	11.65 (10.64–12.76)	10.38 (9.43–11.42)	-15.7*
Sex							
Female	7.48	7.77	7.63	6.68	5.91	6.08	-18.7
Male	16.94	17.35	17.69	18.46	17.66	15.19	-10.3
Age category							
0–34 years	0.21	0.57	0.56	0.64	0.53	0.38	+81.0
35–54 years	10.04	9.64	9.24	9.04	9.12	8.05	-19.8
55–74 years	36.83	35.09	36.54	38.15	34.07	31.00	-15.9
>75 years	51.43	60.21	58.31	51.18	50.64	44.94	-12.6
**Major**							
***Diabetes Population***							
Crude Rate (95% CI)	2.18 (1.92–2.47)	2.10 (1.86–2.37)	1.92 (1.70–2.17)	1.54 (1.35–1.75)	1.54 (1.36–1.74)	1.20 (1.05–1.37)	-45.0*
***General Population***							
Crude Rate (95% CI)	6.20 (5.47–7.03)	6.39 (5.65–7.22)	6.23 (5.51–7.04)	5.31 (4.66–6.05)	5.68 (5.02–6.44)	4.74 (4.14–5.43)	-23.5*
Age-Sex Rate (95% CI)#	6.26 (5.53–7.08)	6.39 (5.65–7.22)	6.18 (5.45–7.00)	5.27 (4.60–6.02)	5.60 (4.90–6.37)	4.61 (3.99–5.32)	-26.4*
Sex							
Female	4.63	3.98	3.93	3.31	3.14	2.81	-39.3*
Male	7.78	8.80	8.53	7.31	8.23	6.68	-14.1
Age category							
0–34 years	0	0.05	0.15	0.20	0.24	0.19	NA
35–54 years	3.05	3.07	2.65	2.85	2.55	1.79	-41.3
55–74 years	16.73	17.12	16.36	13.29	14.62	13.78	-17.6
>75 years	44.02	44.48	44.72	36.56	40.09	29.28	-33.5*

95% CI: 95% Confidence Intervals

^ Chi-squared tests of trend used unless otherwise indicated

# Rates are standardised to the age and sex distribution of the general resident population in the year 2006 and presented as per 100,000 person-years of the general Queensland resident population

* *p* < 0.05.

Table 3 also displays the age-sex standardised incidence (95% CI) in the general population which also decreased from 2005 to 2010 for all outcomes per 100,000 person-years: 23.3% for hospital admission (105.1 (102.0–108.3) in 2005 to 80.6 (77.9–83.4) in 2010), 19.5% for hospital bed days used (1,122 (1,112–1,132) in 2005 to 903 (894–912) in 2010), 19.3% for total amputations (18.57 (17.28–19.95) in 2005 to 14.99 (13.84–16.24) in 2010), 26.4% for major amputations (6.26 (5.53–7.08) in 2005 to 4.61 (3.99–5.32) in 2010), and 15.7% for minor amputations (12.32 (11.28–13.46) in 2005 to 10.38 (9.43–11.42)) (*p* < 0.01 respectively).

Table 4 reports on the results for the evaluation of time trends for each outcome based on the Poisson regression models fitted with and without adjustment for age and sex categories using calendar year as a categorical variable. Age-sex adjusted hospital admission rate ratios (95% CI) demonstrated increases in 2006 (1.059 (1.014–1.104)) and decreases in 2009 (0.880 (0.843–0.919)) and 2010 (0.771 (0.738–0.807)), compared to the referent year of 2005 (*p* < 0.01 respectively). Age-sex adjusted hospital bed days used rate ratios demonstrated decreases in every calendar year compared to the referent year of 2005 (*p* < 0.01 respectively). Results of age-sex adjusted models for all amputation outcomes showed significant rate ratio decreases in 2010 compared to the referent year of 2005; total amputations 0.812 (0.731–0.902)), minor amputations 0.847 (0.746–0.962), and, major amputations 0.744 (0.619–0.895) age-sex adjusted rate ratios in 2010 (*p* < 0.02 respectively).

**Table 4:** Rate ratios of hospitalisation (admissions and occupied bed days) and amputation (total, minor and major) cases in Queensland from 2005 to 2010 with and without adjustment for sex, age and sex*age groups: results from Poisson regression models using calendar year as a categorical variable.

Outcome	Model	2005#	2006	2007	2008	2009	2010
			RR (95% CI)	RR (95% CI)	RR (95% CI)	RR (95% CI)	RR (95% CI)
**Hospitalisation**							
**Admissions**	**1 (nil adjustment)**	Referent	1.069 (1.024–1.115)*	0.994 (0.952–1.037)	0.977 (0.936–1.019)	0.898 (0.859–0.937)*	0.794 (0.759–0.829)*
	**2 (sex + age)**	Referent	1.059 (1.014–1.104)*	0.978 (0.0937–1.021)	0.959 (0.919–1.001)	0.880 (0.843–0.919)*	0.771 (0.738–0.807)*
	**3 (sex + age + sex** * **age)**	Referent	1.059 (1.014–1.104)*	0.978 (0.0937–1.021)	0.959 (0.919–1.001)	0.880 (0.843–0.919)*	0.771 (0.738–0.807)*
**Bed Days**	**1 (nil adjustment)**	Referent	0.961 (0.948–0.974)*	0.976 (0.964–0.990)*	0.958 (0.946–0.970)*	0.899 (0.888–0.912)*	0.834 (0.822–0.845)*
	**2 (sex + age)**	Referent	0.951 (0.939–0.965)*	0.962 (0.949–0.974)*	0.942 (0.929–0.954)*	0.883 (0.871–0.895)*	0.811 (0.800–0.822)*
	**3 (sex + age + sex** * **age)**	Referent	0.951 (0.939–0.965)*	0.962 (0.949–0.974)*	0.942 (0.929–0.954)*	0.883 (0.871–0.895)*	0.811 (0.800–0.822)*
**Amputations**							
**Total**	**1 (nil adjustment)**	Referent	1.029 (0.930–1.140)	1.026 (0.927–1.135)	0.971 (0.877–1.076)	0.949 (0.857–1.051)	0.835 (0.752–0.928)*
	**2 (sex + age)**	Referent	1.019 (0.920–1.129)	1.010 (0.913–1.117)	0.954 (0.862–1.057)	0.931 (0.840–1.031)	0.812 (0.731–0.902)*
	**3 (sex + age + sex** * **age)**	Referent	1.019 (0.920–1.129)	1.010 (0.913–1.117)	0.954 (0.862–1.057)	0.931 (0.840–1.031)	0.812 (0.731–0.902)*
**Minor**	**1 (nil adjustment)**	Referent	1.028 (0.908–1.166)	1.037 (0.916–1.174)	1.029 (0.910–1.165)	0.966 (0.852–1.094)	0.871 (0.766–0.989)*
	**2 (sex + age)**	Referent	1.019 (0.899–1.154)	1.021 (0.902–1.156)	1.011 (0.893–1.144)	0.947 (0.836–1.073)	0.847 (0.746–0.962)*
	**3 (sex + age + sex** * **age)**	Referent	1.019 (0.899–1.154)	1.021 (0.902–1.156)	1.011 (0.893–1.144)	0.947 (0.836–1.073)	0.847 (0.746–0.962)*
**Major**	**1 (nil adjustment)**	Referent	1.030 (0.864–1.228)	1.004 (0.843–1.197)	0.856 (0.714–1.026)	0.917 (0.767–1.094)	0.765 (0.634–0.920)*
	**2 (sex + age)**	Referent	1.019 (0.856–1.215)	0.988 (0.829–1.178)	0.841 (0.701–1.008)	0.899 (0.754–1.074)	0.744 (0.619–0.895)*
	**3 (sex + age + sex** * **age)**	Referent	1.019 (0.856–1.215)	0.988 (0.829–1.178)	0.841 (0.701–1.008)	0.899 (0.754–1.074)	0.744 (0.619–0.895)*

* *p* < 0.05

# Referent year

RR: Rate ratio; 95% CI: 95% Confidence Intervals

Table 5 reports the results for the independent variables of continuous calendar year, sex, age and sex*age groups from the three regression models for each outcome. Results of these models adjusted for age, sex and sex*age interaction variables demonstrated a rate ratio reduction per year for all outcomes; hospital admissions 0.949 (0.942–0.956), hospital bed days used 0.964 (0.962–0.966), total amputations 0.962 (0.946–0.979), minor amputations 0.970 (0.950–0.991) and major amputations 0.945 (0.917–0.974) (*p* < 0.01 respectively).

**Table 5:** Rate ratios of hospitalisation (admissions and bed days) and amputation (total, minor and major) cases in Queensland from 2005 to 2010 with and without adjustment for sex, age and sex*age groups: results from Poisson regression models using calendar year as a continuous variable.

Variables	Hospital Admissions	Hospital Bed Days	Total Amputations	Minor Amputations	Major Amputations
	RR (95% CI)	RR (95% CI)	RR (95% CI)	RR (95% CI)	RR (95% CI)
**Model 1 (nil adjustment)**					
Year	0.953 (0.946–0.961)*	0.969 (0.967–0.970)*	0.967 (0.950–0.983)*	0.975 (0.955–0.996)*	0.949 (0.921–0.978)*
**Model 2 (sex + age)**					
Year	0.949 (0.942–0.956)*	0.964 (0.962–0.966)*	0.962 (0.946–0.979)*	0.970 (0.950–0.991)*	0.945 (0.917–0.974)*
Male	2.200 (2.142–2.259)*	2.168 (2.150–2.185)*	2.608 (2.445–2.782)*	2.689 (2.482–2.910)*	2.455 (2.196–2.745)*
Age: >75 years^	128.676 (117.693–140.684)*	183.328 (177.745–189.087)*	161.065 (127.596–203.313)*	119.040 (91.100–155.550)*	303.958 (187.565–492.581)*
Age: 55–74 years^	67.636 (61.950–73.845)*	82.352 (79.866–84.916)*	81.648 (64.869–102.766)*	73.659 (56.678–95.726)*	108.902 (67.303–176.212)*
Age: 35–54 years^	14.025 (12.802–15.365)*	18.211 (17.645–18.795)*	19.227 (15.181–24.351)*	19.301 (14.754–25.249)*	18.982 (11.553–31.186)*
**Model 3 (sex + age + sex** * **age interaction)**					
Year	0.949 (0.942–0.956)*	0.964 (0.962–0.966)*	0.962 (0.946–0.979)*	0.970 (0.950–0.991)*	0.945 (0.917–0.974)*
Male	1.136 (0.955–1.350)	1.209 (1.138–1.284)*	1.716 (1.071–2.750)*	1.580 (0.929–2.685)	2.317 (0.816–6.576)
Age: >75 years^	87.014 (76.227–99.328)*	131.341 (125.371–137.596)*	124.272 (84.283–183.236)*	84.023 (54.334–129.934)*	301.356 (124.094–731.828)*
Age: 55–74 years^	40.346 (35.386–46.001)*	51.567 (49.228–54.017)*	55.076 (37.431–81.039)*	46.742 (30.418–71.826)*	91.744 (37.706–223.224)*
Age: 35–54 years^	11.227 (9.798–12.864)*	14.686 (14.001–15.404)*	17.110 (11.526–25.401)*	15.561 (10.026–24.153)*	23.928 (9.666–59.234)*
Male*Age >75 years^^	1.879 (1.571–2.248)*	1.701 (1.599–1.811)*	1.456 (0.896–2.366)	1.668 (0.960–2.897)	1.007 (0.350–2.898)
Male*Age 55–74 years^^	2.254 (1.888–2.690)*	2.076 (1.951–2.208)*	1.755 (1.085–2.840)*	1.929 (1.121–3.319) *	1.266 (0.440–3.646)
Male*Age 35–54 years^^	1.453 (1.209–1.746)*	1.426 (1.338–1.519)*	1.188 (0.726–1.946)	1.383 (0.793–2.410)	0.702 (0.237–2.075)

* *p* < 0.05

^Referent Age Category: 0–34 years

^^Referent: Female*Same Age Category

RR: Rate ratio; 95% CI: 95% Confidence Intervals

## Discussion

This population-based study reports the incidence of foot-related hospitalisation and amputation amongst persons with diabetes reduced between 2005 and 2010 in the state of Queensland, Australia. These reductions occurred across all foot-related hospitalisation and amputation outcomes amongst persons with diabetes using both the diabetes (at risk) and general populations as denominators. They also decreased across most age and sex categories over this period, except for the youngest age category (0–34 years). In the general population, incidence of these outcomes initially increased from 2005 levels before stabilising and decreasing to the lowest reported levels in 2010. Absolute numbers of these outcomes amongst persons with diabetes were also fewer in 2010 compared to 2005. Median age and sex proportions of those hospitalised with diabetes foot-related complications remained stable throughout the period, except for a small change in the proportion of males undergoing amputation. The proportionate burden of foot-related hospitalisation amongst persons with diabetes, during this six-year period equated to 0.26% of all hospital admissions (9,474,073) and 0.92% of all bed days used (28,397,347) in Queensland hospitals [34].

Reporting of amputation rates amongst persons with diabetes are prone to many confounding factors including differences in data capture and ascertainment, numerator and denominator definitions, and different crude and standardised rates [3, 12, 15, 16]. It is commonly recommended that to detect a real improvement in amputation rates amongst persons with diabetes in a geographical region, absolute numbers and incidence of amputations should reduce [12, 15, 16]. The findings of this study have demonstrated a reduction in absolute numbers and incidence of amputation amongst persons with diabetes when comparing 2005 and 2010 outcomes. Furthermore this study demonstrated reductions in both the age-sex standardised incidence of the general population, reported as a more conservative measure by many authors, as well as using the crude incidence in the diabetes population [16, 17]. This study, consistent with other studies, reports a much more dramatic reduction using the diabetes population then when using the general population as the denominator [16, 17, 20]. A potential reason for this is evident when comparing the 56% increase in people diagnosed with diabetes [31] with the 12% increase in the general resident population of Queensland [30] over the six-year period of the study. Thus, the authors consider the amputations per diabetes population rate reduction may be exaggerated by the rapid registration of people diagnosed with diabetes in this period and suggest the real reduction may be closer to the findings of the more conservative age-sex standardised incidence in the general population [16, 17].

The reductions in amputation and hospitalisation rates were also experienced across most age and sex categories. The only category that experienced an increase in these outcomes was the youngest age category of 0–34 years. It could be hypothesised that these increases may indicate increasing numbers of younger people experiencing major diabetes foot-related complications consistent with reported increases in numbers of younger people being diagnosed with type 2 diabetes in Australia [35, 36]. Otherwise, consistent with similar studies, amputation and hospitalisation rates increased as age categories increased and males had a 2–3 fold risk in comparison to females [18, 20, 21]. However, interestingly the rate reduction over the six-year period in this study was much greater in females than males. With different rate changes in different age and sex categories, further research is required to investigate the impacts of age and sex on diabetes-related foot complications to assist the targeting of future diabetes health policy.

In the general population, the incidence of foot-related amputation and hospitalisation outcomes amongst persons with diabetes initially increased in 2006 or 2007 before decreasing to the lowest reported levels for the period in 2010. Whilst these improved rates of diabetes foot-related outcomes in the latter part of the period are not likely to be the result of one systematic change, they did coincide with the implementation of multi-faceted ambulatory diabetes foot-related complication services across Queensland in 2008 [27]. These strategies, based on national and international diabetes foot guidelines [2, 37–40], included establishing ambulatory multi-disciplinary diabetic foot teams, increasing use of podiatrists, implementing best practice clinical pathways, clinical training, telehealth expert support and measuring key diabetes foot-related complication clinical performance indicators [27]. The pilot regions initially implementing these strategies in 2008 reported significant improvements in the evidence based treatment of larger volumes of people with diabetes foot-related complications and significant reductions in diabetes foot-related hospitalisation and amputation rates [27, 28]. These strategies were rolled out across Queensland in 2009 and 2010 with the provision of a clinical incentive payment for regions implementing the strategies [28]. In 2010 two thirds of Queensland health regions had implemented these ambulatory service improvements collecting regular data on over 1,800 patients with diabetes foot-related complications [28].

Whilst these multi-faceted ambulatory service changes could be hypothesised to have had an impact on diabetes foot-related hospitalisation and amputation, as reported in other similar studies [16–21], it could equally be hypothesised that other service changes in diabetes care may have also been associated with the reductions. These other service areas reporting improvements in diabetic foot outcomes include tighter metabolic control of patients with diabetes in primary care, improvements in limb-saving vascular procedures and increased medical management of severe infection in tertiary inpatient care [16–21]. Although, there have been no published systematic service changes in these other areas of diabetic foot management in Queensland during the investigated period these hypotheses cannot be discounted. Regardless of any hypothesised cause, large reductions in amputation rates have been reported to occur more readily in populations with very high preceding amputation rates [41]. As per the initial findings of this study, Australia had very high total amputation rates amongst persons with diabetes in the general population in comparison to other nations [4, 13, 22, 23] and these rates were increasing [7, 24, 25]. Thus, any improvements in an Australian population may show more dramatic effects than in regions with lower preceding amputation rates amongst persons with diabetes.

The trend of amputation incidence reduction over the six-year period of this study appears to be mirrored by a corresponding reduction in foot-related hospitalisation amongst persons with diabetes. To date there have been very few population-based studies reporting both foot-related hospitalisation and amputation data amongst persons with diabetes [19]. Thus, this study provides useful population-based findings to begin to quantify the population burden of foot-related hospitalisation and amputation amongst person with diabetes. The hospitalisation reduction reported in this study in conjunction with increased lengths of stay and a reduction in ‘prophylactic’ minor amputation procedures [14, 42], potentially adds to a hypothesis that any improvements in diabetic foot care occurred prior to hospital inpatient care [19].

The impact of reductions in foot-related hospitalisation and amputation not only have benefits on population-based morbidity, but have potential significant functional, quality of life and mortality benefits for those persons with diabetes foot-related complications [5, 8–11] and economic benefits for the wider regional health system [6–7]. Nonetheless, it is recommended that future longitudinal studies are undertaken to determine the state and national amputation rates amongst persons with diabetes in Australia and the wider potential benefits of foot-related hospitalisation and amputation reductions in Australia.

### Strengths and Limitations

This study has many strengths. Firstly, the study is based on a very large representative population of Australia and uses an international standard hospital discharge dataset to determine the outcome numerator [29]. Secondly, it reports absolute numbers and incidence in both the estimated registered diabetes (‘at risk’) population and the official general (‘conservative’) resident population. However, the diabetes population numbers used were an estimate from an official national source and are thus their accuracy is uncertain. Thirdly, this study presents incidence rates, adjusted for age and sex, for the general population and analysed incidence rates using robust regression models. Finally, it is one of the few population-based studies to report both foot-related hospitalisation and amputation rates amongst persons with diabetes to more completely determine a large geographical region’s diabetic foot-related hospitalisation burden and trends. Thus, the findings of this study can be considered to be widely applicable in an Australian context and the aligned hospitalisation and amputation data of interest to the international diabetic foot community.

This study also has a number of limitations. Firstly, as per similar studies, this study can only hypothesise the most likely causes for any reductions, but it cannot conclude any causal associations. Secondly, the study is reliant on retrospective data from only one hospital discharge data source and hospitalisation and amputation cases have not been verified with other data sources for case ascertainment accuracy. However, previous Queensland studies report very high accuracy for amputation case ascertainment using this hospital discharge dataset [23, 43]. Thirdly, Australian ICD-10-AM coding and classification versions are updated biannually and can impact on hospital discharge data collected [44]. However, principal diagnosis codes for diabetes complications were reported to remain consistent throughout this period, except for a change in interpretation in July 2010 which may have resulted in some underestimation of the data collected for hospital admissions in late 2010 [44]. Fourthly, the study did not exclude amputations or admissions that were trauma or malignancy-related in persons with diabetes and perhaps not attributable to diabetes per se. However, previous Queensland studies report very low proportions of trauma and malignancy-related amputations within total amputations performed [23]. Fifthly, the study did not report on non-diabetes related amputations as a ‘pseudo-control’. However, an Australian study reported all-cause lower extremity amputation rates remained relatively stable for the same period [45], whilst, another Australian state reported amputation rates amongst persons with diabetes in older age groups remained stable and non-diabetes rates reduced [46]. Lastly, the study can only report cases and is unable to differentiate between individuals, and thus, was unable to report on amputations of individuals, new amputees or first and repeat amputation rates.

## Conclusions

This study has reported significant reductions in foot-related hospitalisations and amputations amongst persons with diabetes across a large representative population of Australia. These reductions are consistent with other international studies reporting improvements in amputation and hospitalisation rates that coincided with improvements in diabetic foot care. However, this appears to be the first time statewide reductions of foot-related amputations or hospitalisations amongst persons with diabetes have been reported in Australia. Future research is required to determine the Australian national diabetes foot-related hospitalisation and amputation rate trends and the causal relationships of any service changes.
